# Subterranean Clover Stunt Virus Revisited: Detection of Two Missing Genome Components

**DOI:** 10.3390/v11020138

**Published:** 2019-02-04

**Authors:** Dennis Knierim, Quentin Barrière, Ioana Grigoras, Stephan Winter, Heinrich-Josef Vetten, Mark Schwinghamer, John Thomas, Paul Chu, Bruno Gronenborn, Tatiana Timchenko

**Affiliations:** 1 Deutsche Sammlung von Mikroorganismen und Zellkulturen GmbH (DSMZ), Inhoffenstraße 7B, 38124 Braunschweig, Germany; dennis.knierim@gmail.com (D.K.); stephan.winter@dsmz.de (S.W.); 2 Institute for Integrative Biology of the Cell, UMR9198, CNRS, Université Paris-Sud, CEA, 91198 Gif-sur-Yvette, France; quentin.barriere@i2bc.paris-saclay.fr (Q.B.); ioana.popescu@issb.genopole.fr (I.G.); bgronenborn@gmail.com (B.G.); 3 Im Spargelfeld 1, 38162 Cremlingen, Germany; vettenjosef8@gmail.com; 4 NSW Department of Primary Industries, Tamworth Agricultural Institute, 4 Marsden Park Road, Calala, NSW 2340, Australia; 5 The University of Queensland, QAAFI, Ecosciences Precinct, GPO Box 267, Brisbane, QLD 4001, Australia; j.thomas2@uq.edu.au; 6 Institute of Microbiology, Chinese Academy of Sciences, Beijing 100101, China; 7 22 Megalong Crescent, Harrison, ACT 2914, Australia; paul@genomicsproducts.com.au

**Keywords:** nanovirus, virus evolution, rolling circle replication, high-throughput sequencing

## Abstract

Subterranean clover stunt virus (SCSV) is a type species of the genus *Nanovirus* in the family *Nanoviridae*. It was the first single-stranded DNA plant virus with a multipartite genome, of which genomic DNA sequences had been determined. All nanoviruses have eight genome components except SCSV, for which homologs of two genome components present in all other nanovirus genomes, DNA-U2 and DNA-U4, were lacking. We analysed archived and more recent samples from SCSV-infected legume plants to verify its genome composition and found the missing genome components. These results indicated that SCSV also has eight genome components and is a typical member of the genus *Nanovirus*.

## 1. Introduction

Nanovirids, members of the genera *Babuvirus* and *Nanovirus* of the *Nanoviridae* family, are plant viruses with a genome consisting of multiple circular single-stranded (ss) DNA molecules that are individually encapsidated in small isometric particles of about 20 nm in diameter [1]. These viruses are transmitted in a circulative persistent (non-propagative) manner by various aphid species [2]. Currently, there are eight species approved by the International Committee on Taxonomy of Viruses in the Genus *Nanovirus*: *Black medic leaf roll virus* [3], *Faba bean necrotic stunt virus* [4], *Faba bean necrotic yellows virus* [5,6], *Faba bean yellow leaf virus* [7], *Milk vetch dwarf virus* [8], *Pea necrotic yellow dwarf virus* [9], *Pea yellow stunt virus* [3], and *Subterranean clover stunt virus* [10]. Subterranean clover stunt virus (SCSV) is the type species of the genus *Nanovirus* [1,11] as it is the first nanovirus of which genome components have been sequenced [12]. Recently, two new nanovirus species have been discovered, one from the wild perennial legume *Sophora alopecuroides* L. in Iran [13] and another one from *Vicia cracca* L. in France [14], providing further evidence for *Fabaceae* being the principal hosts of nanoviruses.

With the exception of SCSV, the genomes of all nanoviruses, including those of the two recently described nanoviruses Sophora yellow stunt associated virus (SYSaV) [13] and cow vetch latent virus (CvLV) [14] comprise eight characteristic ssDNA circles of about 1 kb in size. The genome components are named according to the functions of the respective encoded proteins: DNA-R (master replication initiator protein, M-Rep, required for replication initiation of all genomic DNAs) [15], DNA-S (structural = capsid protein, CP) [5], DNA-C (cell cycle link protein, Clink, modulates the host’s cell cycle in favour of nanovirus replication) [16], DNA-M (movement protein, MP) and DNA-N (nuclear shuttle protein, NSP). The functional identities of MP and NSP were inferred from similarities with the corresponding geminivirus proteins [17,18,19]. In addition, three other DNA molecules, DNA-U1, DNA-U2 and DNA-U4, are typical constituents of a nanovirus genome, however, the function of the proteins they encode are hitherto not known. Only interaction data of the U1 and U4 proteins with other nanovirus proteins and subcellular localization data are available [20]. The U1 and U2 proteins of faba bean necrotic stunt virus (FBNSV) are essential symptom determinants whereas the U4 protein is dispensable for symptom formation and aphid transmission [21]. The NSP is also not required for FBNSV symptom development but is obligatory for the spread of the virus as it acts as mandatory helper factor for nanovirid aphid transmission [21]. Moreover, the NSP of pea necrotic yellow dwarf virus has been shown to interact with a plant G3BP-like protein, a constituent of stress granules [17].

Despite the wealth of biological data available for the subterranean clover stunt disease and its causative virus [22,23,24,25,26,27,28], SCSV remained the sole nanovirus with only six integral genome components, lacking DNA-U2 and DNA-U4 [1,10,15]. This prompted us to elucidate whether SCSV, thus far only found in Australia, might have a genome composition different from those of all other nanoviruses, implying that the two absent components might be dispensable in SCSV infections.

In this study, we have detected in various SCSV samples from three different hosts the missing genome components DNA-U2 and DNA-U4, providing evidence that SCSV has eight genome components, the same as all currently known members of the genus *Nanovirus*.

## 2. Materials and Methods

### 2.1. Virus Isolates and Field Materials

SCSV isolate F [29] and SCSV isolate SCS1 (unpublished) isolated from field-infected subterranean clover (*Trifolium subterraneum*) were maintained in the same host for several years by successive transmissions at CSIRO, Canberra, using the cowpea aphid (*Aphis craccivora*) [12] and were provided in 2001 to H.-J.V. In this study, the SCSV F provided in 2001 is designated SCSV-[AU;F*] to differentiate it from the SCSV F isolate (designated as SCSV-[AU;F]) that was sampled in 1992 and whose genome sequence had been published in 1995 [10]. SCS1 is designated as SCSV-[AU;SCS1].

Two additional SCSV-infected field samples collected and deposited by the late M. Schwinghamer in the New South Wales Plant Pathology Herbarium (DAR), Orange, NSW, were SCSV-[AU;2534B] from faba bean (*Vicia faba*) (DAR collection number 76607), collected at Myall Vale in 1997, and SCSV-[AU;3771A] from pea (*Pisum sativum*) (DAR collection number 76849), collected at Quandialla in 2003. They were sent in 2012 to B.G. as dried leaf tissue.

To complement analyses of these archived samples, we collected in December 2018 at Harrison, ACT, seventy-two field samples of subterranean clover and checked fourteen of them for the presence of SCSV. SCSV from these samples is designated SCSV-[AU;Har].

### 2.2. DNA Extraction, RCA, PCR, Cloning, and Sequencing

Total DNA was extracted from SCSV-infected dried tissue according to a modified Edwards protocol as described previously [4] with an additional step of phenol-chloroform extraction. Total DNA preparations were subjected to rolling circle amplification (RCA) to increase the amount of circular DNA using an Illustra TempliPhi Amplification Kit (GE Healthcare, Little Chalfont, Buckinghamshire, UK) following the manufacturer’s instructions. The isothermal incubation for RCA was at 30 °C for 18–20 h followed by a final incubation at 65 °C for 10 min.

RCA products were used as templates for PCR amplification of SCSV genome components using different combinations of primers (Supplementary Table S1) and Phusion DNA polymerase (Thermo Fisher, Waltham, MA, USA) or Taq II DNA polymerase (Eurobio Ingen, Les Ulis, France). For this, 1 μL of tenfold diluted RCA product was added to 20 or 50 μL of reaction mixture containing 10 pmol each of corresponding primers, 50 μM of each deoxynucleoside triphosphate, and 0.5 units of DNA polymerase in provided reaction buffer. In addition, PCR amplifications using degenerate primers were performed using Taq II DNA polymerase. Amplification conditions are specified in Supplementary Table S1.

PCR products were either sequenced directly using a commercial sequencing provider (Eurofins Genomics, Ebersberg, Germany) or inserted into plasmid pBluescript KSII(+) (pBKSII) (Stratagene/Agilent, Santa Clara, CA, USA) linearized by *Eco*RV or *Hinc*II restriction endonucleases. Insert DNAs of recombinant plasmids were sequenced, and sequences were analysed with DNASTAR Lasergene version 8.0.2 (DNASTAR, Inc. Madison, Wisconsin, USA) and Geneious version 8.1.8 (Biomatters, Auckland, New Zealand).

Libraries for deep sequencing were prepared from diluted (1:10 *v*/*v*) RCA products using the Nextera XT Library Kit (Illumina, San Diego, CA, USA). Quality checked libraries were sequenced using Illumina MiSeq (paired end reads 2 × 301 bp) (DSMZ, Braunschweig, Germany).

### 2.3. Sequence Assembly and Analysis

Illumina reads were trimmed, and de novo assembled using the Geneious assembler v. 10.2.3 (assembler settings: Low Sensitivity/Fast and circularized contigs). Local BlastN and BlastP against the plant virus reference databank from the National Center for Biotechnology Information (accessed on 11 June 2018) identified contigs matching nanovirus sequences. To generate the final consensus sequence for the genome molecules from a given isolate, all Illumina reads of the respective isolate were mapped in a single step to all its genome components (Low Sensitivity/Fastest [10%]).

Multi-alignments were done using MUSCLE [30] as implemented in Geneious or MEGA7 [31]. Phylogenetic analyses were conducted in MEGA7 and SDT v. 1.2 [32]. Maximum likelihood trees (100 bootstrap repetitions) were constructed using MEGA7 or PhyML [33], with nucleotide substitution models chosen by model test in MEGA7.

## 3. Results

### 3.1. Identification of SCSV DNA-U2 and DNA-U4

Alignment of the DNA-U2 and DNA-U4 sequences of all nanoviruses known in 2014 [3] indicated small regions of similarity in their respective coding parts (Supplementary Figures S1 and S2). This allowed the design of the degenerate primers nanoU2dir and nanoU4dir (Supplementary Table S1). Stem-loop regions of nanovirus DNA-U2 and DNA-U4 share longer stretches of sequence identity, which allowed the design of the primers nanoSTLdir and nanoSTLrev. We used these primers to potentially amplify SCSV DNA-U2 and DNA-U4 by gradient PCR with RC-amplified template DNA derived from isolates SCSV-[AU;3771A] Quandialla 2003 from pea and SCSV-[AU;2534B] Myall Vale 1997 from the faba bean.

**Figure 1:**
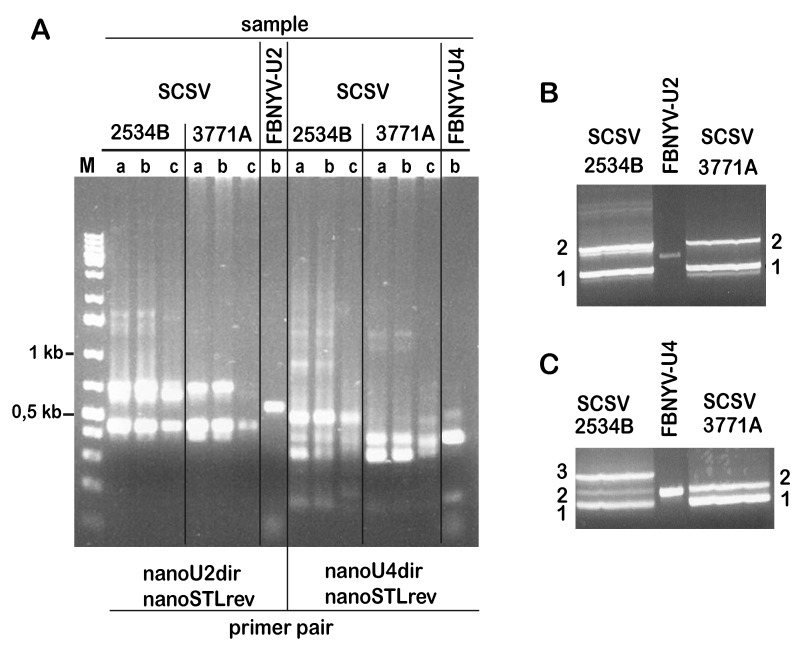
Amplification of subterranean clover stunt virus (SCSV) DNA-U2 and DNA-U4 fragments by PCR. (**A**) Gel electrophoresis of PCR products obtained from different nanovirus samples (indicated at the top) using primer nanoSTLrev and the degenerate primers specific for DNA-U2 (nanoU2dir) or DNA-U4 (nanoU4dir), as indicated at the bottom. Letters a, b, and c indicate annealing temperatures of the PCR reactions: 45.0, 53.7 and 65.0 °C respectively; (**B**) preparative gel-electrophoresis of the same PCR products as in (A) for DNA-U2 amplification; (**C**) preparative gel-electrophoresis of the same PCR products as in (A) for DNA-U4 amplification.

Two major amplification products were obtained from the DNA samples of these isolates when targeting DNA-U2 (Figure 1A). By contrast, amplifications aimed at identifying DNA-U4 yielded three products for isolate 2534B and two products for isolate 3771A, the proportion of the latter differed slightly depending on the annealing temperature during PCR. Only amplifications at three different annealing temperatures are shown in Figure 1A. Products obtained at different annealing temperatures from the respective isolates were pooled, size-separated by agarose gel electrophoresis (Figure 1B,C), gel-eluted, and directly sequenced using the same primers as for PCR.

The sequences of the amplification products marked “2” (Figure 1B) for DNA from both isolates were similar to published DNA-U2 sequences of other nanoviruses. By contrast, the sequences of the PCR-products of both isolates, marked “1” in Figure 1B, corresponded to the SCSV DNA-R sequence. The sequences of the PCR products from both isolates, marked “1” in Figure 1C, had similarity with published DNA-U4 sequences of nanoviruses. In contrast, the sequences of the products marked “2” (both isolates) and “3” (isolate 2534B only) all corresponded to SCSV DNA-C sequences. Based on the PCR-amplified DNA-U2 and DNA-U4 sequences we designed component- specific back-to-back primers (Supplementary Table S1) and used them to amplify full-length DNA-U2 and DNA-U4 of SCSV. This way we identified, cloned and sequenced DNA-U2 and DNA-U4 from isolates SCSV-[AU;3771A] and SCSV-[AU;2534B].

In addition, we designed specific primers for the thus far known SCSV components DNA-R, DNA-S, DNA-C, DNA-M, DNA-N, DNA-U1, based on sequences of SCSV-[AU;F] [10,34] (Supplementary Table S1). We used these primers to amplify the respective genome components of isolates SCSV-[AU;3771A] and SCSV-[AU;2534B], which we subsequently cloned and sequenced.

By the same way as described for SCSV-[AU;3771A] and SCSV-[AU;2534B] we also obtained a complete copy of DNA-U2 from isolate SCSV-[AU;F*]. However, we never obtained any PCR products from SCSV-[AU;F*] DNA when using the various DNA-U4 primers listed in Supplementary Table S1. Remarkably, using RC-amplified DNA derived from another archived SCSV isolate, SCSV-[AU;SCS1], which had also been maintained in subterranean clover for a prolonged period, we did not detect any DNA-U4 either when using the U4-specific primers of Table S1 whereas other DNAs (e.g., DNA-U2, DNA-R, DNA-S) were detected by PCR.

### 3.2. DNA Sequence Analysis and Comparison of Three Different SCSV Isolates

For an unbiased analysis of the SCSV genome and to assure that we did not miss any SCSV and SCSV-associated DNAs in our PCR and cloning experiments, RCA DNA from all three isolates were subjected to MiSeq sequencing, complementing any previous information obtained by Sanger-sequencing of the cloned eight integral nanovirus genome components of SCSV-[AU;3771A]. Thus, eight nanovirus genomic DNA components were identified for SCSV isolates Quandialla 2003 (SCSV-[AU;3771A]) and Myall Vale 1997, SCSV-[AU;2354B] while RCA preparations of SCSV-[AU;F*] did not contain any DNA-U4. The number of reads and the average coverage per genome component by deep sequencing of DNA from the three SCSV isolates are shown in Supplementary Table S2.

**Figure 2:**
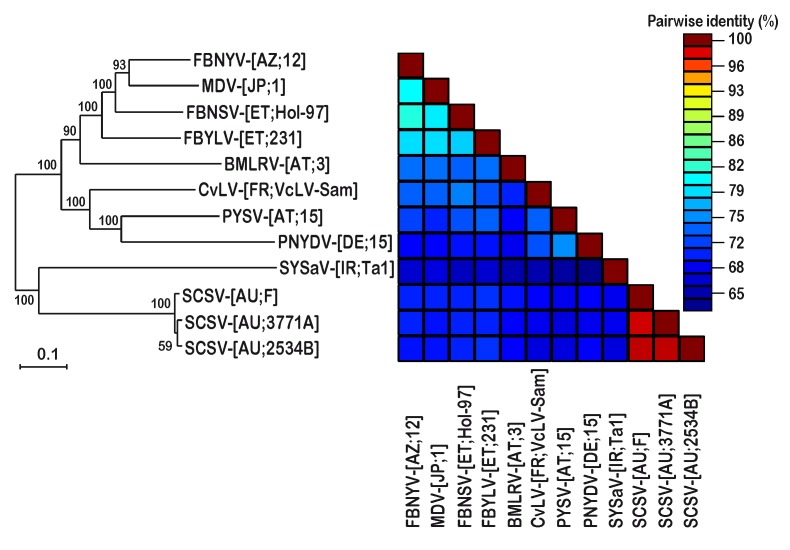
Maximum likelihood tree of nanovirus genome sequences. Individual genomic DNAs of faba bean necrotic yellows virus (FBNYV), milk vetch dwarf virus (MDV), faba bean necrotic stunt virus (FBNSV), faba bean yellow leaf virus (FBYLV), black medic leaf roll virus (BLMRV), cow vetch latent virus (CvLV), pea yellow stunt virus (PYSV), pea necrotic yellow dwarf virus (PNYDV), Sophora yellows associated virus (SYSaV), and subterranean clover stunt virus (SCSV) were concatenated in the order DNA-R, -S, -C, -M, -N, -U1, -U2, -U4 and aligned by MUSCLE. The tree was constructed in MEGA7 using the GTR+G+I nucleotide substitution model and is midpoint rooted. Branch support is shown as per cent bootstrap values. The scale bar represents 0.1 substitutions per site. On the right, a color-coded Sequence Demarcation Tool (SDT) matrix shows per cent pairwise sequence identities.

The comparison of SCSV genome sequences with those of selected isolates of nanovirus species using concatenated genomic DNAs (in order of DNA-R, -S, -C, -M, -N, -U1, -U2, and -U4) showed sequence differences of 22–37% between them while the genomes of the three SCSV isolates were 97–98% identical (see Supplementary Table S4) and formed a separate clade from other nanovirus sequences upon phylogenetic analysis (Figure 2).

**Figure 3:**
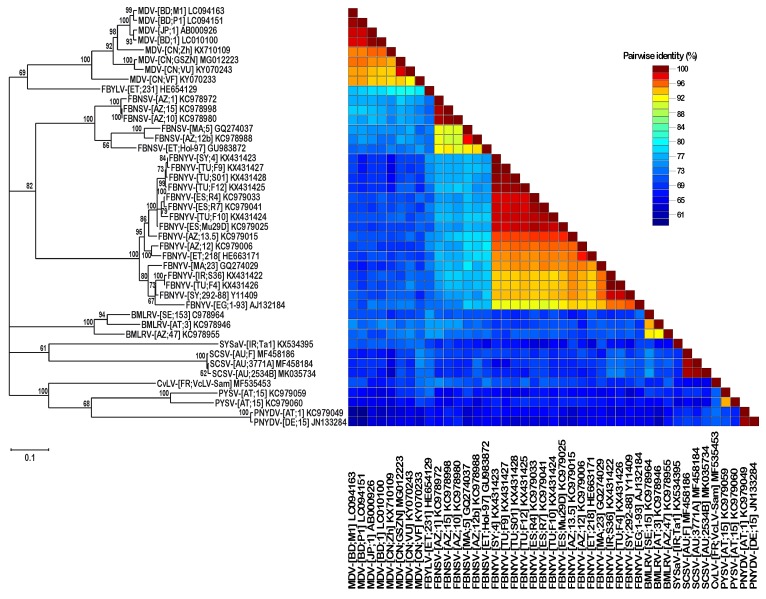
Maximum likelihood tree of nanovirus DNA-U2 sequences. Nanovirus DNA-U2 sequences were aligned by MUSCLE and the tree was constructed in MEGA7 using the TN93+G+I nucleotide substitution model and is midpoint rooted. Branch support is shown as per cent bootstrap values while low support (<50% bootstrap) branches were collapsed. The scale bar represents 0.1 substitutions per site. The right panel shows per cent pairwise sequence identities in a color-coded SDT matrix.

**Figure 4:**
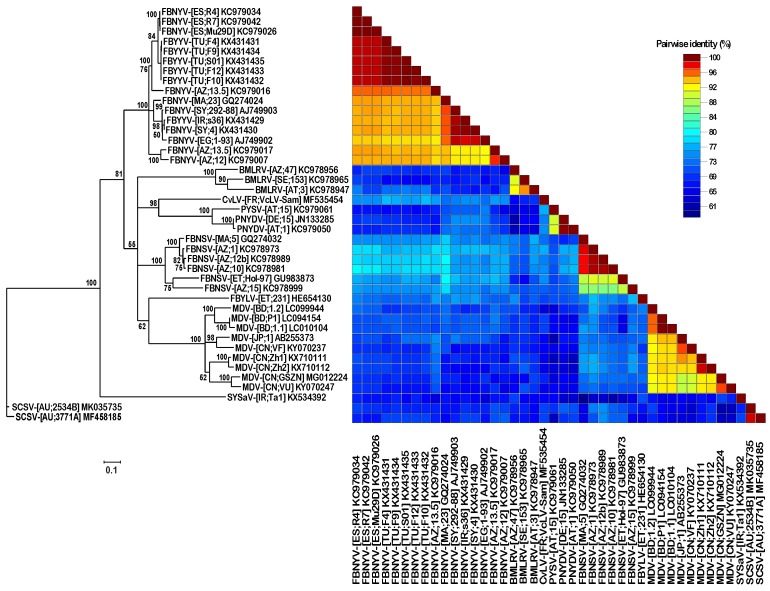
Maximum likelihood tree of nanovirus DNA-U4 sequences. Nanovirus DNA-U4 sequences were aligned by MUSCLE and a midpoint rooted PhyML tree was constructed using the TN93+G+I nucleotide substitution model. Branch support is shown as per cent bootstrap values and low support (<50% bootstrap) branches were collapsed. The scale bar represents 0.1 substitutions per site. The right panel shows per cent pairwise sequence identities in a color-coded SDT matrix.

The SCSV DNA-U2 components identified here had highly similar sequences (98–99% sequence identity) and were clearly distinct (61–70% sequence identity) from the DNA-U2 sequences of all other nanoviruses (Figure 3). Similarly, DNA U4 sequences of SCSV were also distinct from those of other nanoviruses (Figure 4).

Interestingly, a second DNA-U4 molecule (DNA-U4.2; GenBank accession MK035736) was identified by deep sequencing in SCSV-[AU;2534B] (Table 1). It is eight nucleotides shorter and represents a recombination event, with nucleotides 934 to 948 of DNA-U4.2 being derived from DNA-C (or DNA–M or DNA-U1) of the same isolate (Supplementary Figure S3). In addition, deep sequencing of DNA of SCSV isolate [AU;3771A] uncovered an additional recombinant DNA-M, showing a recombination event with DNA-R at nucleotide 927 to 958 (Supplementary Figure S4). In both cases, the genome components DNA-M.1 and DNA-M.2 (recombinant, GenBank accession MK291271) and DNA-U4.1 and DNA-U4.2 (recombinant) were about equally abundant (see Supplementary Table S2). Remarkably, the DNA-N molecule identified by deep sequencing of isolate SCSV-[AU;F*] differed from the original DNA-N of SCSV-[AU;F] [12], available at GenBank (accession U16733), as well as from DNA-N of SCSV-[AU;3771A] and SCSV-[AU;2534B] by a 22 nucleotide deletion (nt 294–316) and a 87 nt recombinant sequence derived from DNA-S (position 896–982) (Supplementary Figure S5). We had found the same deletion in a recombinant DNA-N by an earlier pyro-sequencing of SCSV-[AU;F*] DNA and had verified its physical existence by sequencing a cloned molecule of DNA-N from SCSV-[AU;F*].

An overall comparison of the SCSV-[AU;F] and SCSV-[AU;F*] genome sequences (including the two alpha-satellites) showed a total of 102 changes, counting deletions >20 nt and the recombination as single events (Table S3). This corresponds to a rate of substitutions per site and year of 1.1 × 10exp−3, which fits well with the substitution rate of 1.8 × 10exp−3 per site and year determined for faba bean necrotic stunt virus (FBNSV) [35].

In addition to the eight SCSV genome components, we found in all three SCSV infected plant samples analysed here the two alpha-satellites described earlier [10], formerly named SCSV C2 (GenBank accession U16731) and SCSV C6 (GenBank accession U16735) and reclassified as subterranean clover stunt alpha-satellite 1 and subterranean clover stunt alpha-satellite 2, respectively [36]. A third alpha-satellite (GenBank accession MK291270), clearly distinct from the two aforementioned alpha-satellites but most closely related (77% sequence identity) to Sophora yellow stunt alpha-satellite 3 (GenBank accession KX534406) [13,36], was found associated with SCSV-[AU;3771A].

The absence of DNA-U4 in the historical isolate SCSV-[AU;F*] when analysed by deep sequencing prompted us to analyse more recent samples of subterranean clover to assess any potential influence of this host on DNA-U4 maintenance upon infection. Of the seventy-two samples of subterranean clover displaying SCSV-like symptoms, collected in December 2018, fourteen were randomly selected for PCR analyses. When tested by PCR using SCSV component-specific primers ten out of fourteen samples analysed contained SCSV DNAs, in seven of them all eight integral genome components of nanoviruses, including DNA-U2 and DNA-U4, were detected (see Supplementary Table S5). When RC-amplification preceded the PCR analyses, all eight SCSV components were detected in the ten previously SCSV-positive samples as well as in additional ones with an apparently lower virus content (Supplementary Table S6). We determined the DNA sequences of DNA-U4 components of three different virus isolates from subterranean clover. They were about 96% identical to the DNA-U4 molecules of the SCSV isolates from pea (SCSV-[AU;3771A]) and the faba bean (SCSV-[AU;2534B]) (see Supplementary Table S4).

## 4. Discussion

Using degenerate primers designed on the basis of nanovirus DNA-U2 and DNA-U4 sequences, we were able to amplify and clone the corresponding genome components representing DNA-U2 and DNA-U4 from two SCSV genomes from virus-infected grain legume field samples, SCSV-[AU;2534B] from the faba bean and SCSV-[AU;3771A] from the pea.

A comparison of the SCSV genome with those of other members of the genus *Nanovirus* revealed that the SCSV genome is not so different after all, since it has the same eight integral genome components as other nanoviruses described to date. Like the other members of the genus, SCSV isolates are associated with alpha-satellites. A closer look by deep sequencing uncovered not only the two known alpha-satellites but also a third one, related to Sophora yellow stunt alpha-satellite 3.

We also found several examples of recombinant SCSV genome components, in two cases coexisting with non-recombinant counterparts, e.g., DNA-M.1 and DNA-M.2 in isolate [AU;3771A] as well as DNA-U4.1 and DNA-U4.2 in isolate [AU;2534B]. Another interesting and quite peculiar variant is the single DNA-N molecule found in SCSV-[AU;F*], which combines a 22 nucleotide deletion 5′ of the sequence coding for the transmission factor NSP with a 87 nucleotide recombinant stretch 5′ of the common region stem-loop derived from DNA-S. Deletion and recombination occurred in the noncoding region, leaving the amino acid sequence of the NSP unaffected. At which moment during the passaging of SCSV-F such a quite drastic DNA-N mutant had arisen and became established remains open. Intra- and inter-genome recombination appears common among ssDNA viruses [37] and has been found in all nanovirid genomes analysed for recombination, including SCSV [3,38,39,40,41].

It remains a matter of speculation why no DNA-U2 and DNA-U4 molecules had been found in earlier molecular analysis of SCSV-[AU;F] [10] from subterranean clover. The most trivial explanation would be that in the cloning experiments described [10,29], the authors simply missed these two DNAs, as had also been the case for the essential DNA-R of SCSV [34].

Another possibility would be that upon prolonged SCSV passaging in subterranean clover by aphid transmission [12], DNA-U2 and DNA-U4 were lost. However, that is not true for DNA-U2 since we detected it in SCSV-[AU;F*] by both PCR and deep sequencing. By contrast, an explanation for the absence of DNA-U4 in sample SCSV-[AU;F*] is not as straightforward. Contrary to its presence in the two SCSV genomes from pea (SCSV-[AU;3771A]) and the faba bean (SCSV-[AU;2534B]), we were unable to detect any DNA-U4 in SCSV-[AU;F*], both by DNA-U4-specific PCR analysis as well as by deep sequencing. Hence, DNA-U4 was most probably lost from the initial subterranean clover isolate of SCSV during initial separation from other viruses or during prolonged maintenance of the isolate by repeated aphid transmissions in the laboratory. Similarly, using component-specific PCR we also did not detect any DNA-U4 in SCSV-[AU;SCS1], an isolate that had been maintained in subterranean clover for a longer period in the laboratory.

To rule out the possibility that the subterranean clover host itself was unable to maintain DNA-U4 along with the other SCSV genome components early during the infection process, we collected recent samples of subterranean clover displaying SCSV-like symptoms. At least ten of the fourteen plants analysed were infected by SCSV, with all ten infected plants containing the eight genomic DNAs characteristic of legume-infecting nanoviruses. This clearly shows that field-infected subterranean clover maintains all eight integral genome components at least until the characteristic disease symptoms develop.

Loss of nanovirus genome components upon successive aphid transfer under laboratory conditions has been observed in several instances and has caused the loss of virus isolates; see for instance reference [4]. A similar loss of several isolates had also happened during laboratory maintenance of SCSV (unpublished).

In this context it should be mentioned that another nanovirus, FBNSV, intentionally devoid of DNA-U4, could be maintained for a prolonged period of more than two years by successive aphid transmissions without any obvious difference from the wild-type virus in symptom severity and transmissibility [21]. In addition, the relative abundance of individual genome components of FBNSV varies according to a host-specific genome formula between faba bean (*Vicia faba*) and barrel clover (*Medicago truncatula*) [42]. Yet, the reason for the absence of DNA-U4 in the historical SCSV-[AU;F*] and SCSV-[AU;SCS1] appears to be an unintentional loss from subterranean clover.

Due to the lack of suitable diagnostic tools in early studies, the reservoir hosts of SCSV have not been identified. Among a variety of various leguminous species analysed, the woolly burr medic (*Medicago minima*) appears to be a plausible candidate for a reservoir host as it is preferred over subterranean clover by the cowpea aphid (*A. craccivora*), the principal and most effective vector aphid of SCSV, and also serves as an overwintering host of the vector [25,26]. In addition, the highly susceptible black medic (*Medicago lupulina*) and burr medic (*Medicago hispida*) would represent potential candidates of reservoir hosts [23,43]. Assessing the ecology and epidemiology of SCSV by modern molecular analysis tools may provide attractive new aspects of the subterranean clover stunt disease and its causative agent, the nanovirus SCSV.

## 5. Conclusions

We have shown that SCSV also possesses a genome that comprises eight distinct and integral DNA components, as observed for all members of the genus *Nanovirus*.

## Figures and Tables

**Table 1:** Accession numbers of genome DNAs of subterranean clover stunt virus isolates and their associated alpha-satellites.

DNA		Virus Isolate			
		SCSV-[AU;3771A]	SCSV-[AU;2534B]	SCSV-[AU;F]	SCSV-[AU;F*] ^1^
**Genome components**	**DNA-R**	MF458178	MK035728	AJ290434	
	**DNA-S**	MF458179	MK035729	U16734	
	**DNA-C**	MF458180	MK035730	U16732	
	**DNA-M** **DNA-M.2**	MF458181 ^2^ MK291271	MK035731	U16730	
	**DNA-N**	MF458182	MK035732	U16733	MK291272
	**DNA-U1**	MF458183	MK035733	U16736	
	**DNA-U2**	MF458184	MK035734	-	MF458186
	**DNA-U4** **DNA-U4.2**	MF458185	MK035735 ^3^ MK035736	-	-
**Alphasatellites**	**SCSA 1**	MK291268	MK035737	U16731	
	**SCSA 2**	MK291269	MK035738	U16735	
	**SYSA 3**	MK291270	-	-	

^1^ SCSV-[AU;F*] represents sequences of DNAs after about nine years propagation of the original SCSV-[AU;F] isolate in the laboratory. Only SCSV-[AU;F*] DNAs with >30 differences from SCSV-[AU;F] were deposited in GenBank. All sequences determined in this study are provided as Supplementary data. ^2^ This molecule is named DNA-M.1 for isolate SCSV-[AU;3771A] ^3^ This molecule is named DNA-U4.1 for isolate SCSV-[AU;2534B].

## Supplementary Materials

The following are available online at http://www.mdpi.com/1999-4915/11/2/138/s1, Figure S1: Multialignment of nanovirus DNA-U2 sequences, Figure S2: Multialignment of nanovirus DNA-U4 sequences, Figure S3: Multi-alignment of SCSV DNA-U4 sequences, Figure S4: SCSV-[AU;3771A]_DNA-M.1_MF458181 vs. DNA-M.2_MK291271_alignment, Figure S5: SCSV-[AU;F]_DNA-N_U16733 vs. SCSV-[AU;F*]_DNA-N_MK291272 alignment; Table S1: Primers used for amplification of SCSV DNAs, Table S2: Summary of deep sequencing data from three SCSV samples, Table S3: SCSV nucleotide substitutions, Table S4: Pairwise sequence identities of selected nanovirus genomes and DNAs U2 and -U4, Table S5: Detection by PCR of SCSV DNAs in field samples of symptomatic subterranean clover, Table S6: Detection of SCSV DNAs in field samples after rolling circle amplification followed by PCR; Supplementary text file: DNA sequences determined and used for comparisons, in FastA format.
