# Supplementary material for: Subterranean Clover Stunt Virus Revisited: Detection of Two Missing Genome Components
Source: Viruses. 2019 Feb 4;11(2):138. doi: 10.3390/v11020138 (PMC6410307; doi:10.3390/v11020138)
Supplement: Supplementary file 1 [file viruses-11-00138-s001.zip › Knierim_Supplementary/Knierim_Suppl_FigS1-S5_TableS1-S3,S5,S6.pdf]

```
FBNYV-[ES;R4]_DNA-U2 KC979033        435-GCTAACGAAGAAGCGTTAGGGGAGTTTTGTAGATATCACGGAAGGAGAGTAC-486
FBNYV-[ES;Mu29D]_DNA-U2 KC979025     435-GCTAACGAAGAAGCGTTAGGGGAGTTTTGTAGATATCACGGAAGGAGAGTAC-486
FBNYV-[ES;R7]_DNA-U2 KC979041        435-GCTAACGAAGAAGCGTTAGGGGAGTTTCGTAGATATCACGGAAGGAGAGTAC-486
FBNYV-[AZ;13.5]_DNA-U2 KC979015      435-GCTAACGAAGAAGCGTTAGGGGAGTTTTGTAGATACCATGGAAGGAGAGTAG-486
FBNYV-[MA;23]_DNA-U2 GQ274029        435-GCTAACGAAGAAGCCTTAGGAGAGTTTTGTAGATATCATGGAAGGATAGTAT-486
FBNYV-[SY;292-88]_DNA-U2 Y11409      435-GCTAACGAAGAAGCGTTAGGAGAGTTTTGTAGATATCATGGAAGGATAGTAT-486
FBNYV-[EG;1-93]_DNA-U2 AJ132184      435-GCTAACGAAGAAGCGTTAGGAGAGTTTTGTAGATACCATGGAAGGAGAATAT-486
FBNYV-[AZ;12]_DNA-U2 KC979006        435-GCTAACGAAGAAGCGTTAGGAGAGTTTTGTAGATATCATGGAAGGAGAGTAC-486
FBNSV-[AZ;1]_DNA-U2 KC978972         431-GCCCATGAAGATGTTCTTGGTGAATTCTGCAGACGCCATGGAAGAAGAGTTC-482
FBNSV-[AZ;10]_DNA-U2 KC978980        431-GCCCATGAAGATGTTCTTGGTGAATTCTGCAGACGCCATGGAAGAAGAGTTC-482
FBNSV-[MA;5]_DNA-U2 GQ274037         425-GCATACGAAGATGTTCTTGGAGAATTTTGCAGACGCCATGGAAGAAGAGTCC-476
FBNSV-[ET;Hol-97]_DNA-U2 GU983872    429-GCCCACGAAGATGTTCTTGGTGAATTCTGCAGACTCCATGGAAGAAGAGTTC-480
FBNSV-[AZ;15]_DNA-U2 KC978998        430-GCCCATGAAGATGTTCTTGGTGAATTCTGCAGACGCCATGGAAGAAGAGTTC-481
FBYLV-[ET;231]_DNA-U2 HE654129       445-GCTCACGAAGATGTTCTTGGGGAAATATGCAGATATCATGGAAGAAGAGTTA-496
MDV-[JP;1]_DNA-U2 AB000926           422-CGCAATGAAGATGTTCTTGGAGAGATGTGCAGACGCCATGGAAGGAAATTGC-473
BMLRV-[AT;3]_DNA-U2 KC978945         452-TCCAATGAAGATATGTTGGGTCAGTTCTGCAGACGCCATGGAAGAAGAGTCA-503
BMLRV-[SE;153]_DNA-U2 KC978964       422-TCCAATGAAGATATGCTTGGTCAGTTCTGCAGACGCCATGGAAGAAGGCTCA-473
BMLRV-[AZ;47]_DNA-U2 KC978955        425-TCTAACGAAGATATGTTGGGTCAGTTCTGCAGACGCCATGGAAGAAGGCTCA-476
PYSV-[AT;15]_DNA-U2 KC979059         500-TCAAATGAAGATATGCTTGGCAAGTTCTGCAGACGCCATGGAAGAAGCGTCA-451
PNYDV-[DE;15]_DNA-U2 JN133284        378-TTTAATGAAGATATGGTTGGAATTCGCTGTCGTCGTCATGGAAGAAGAGTGA-440
PNYDV-[AT;1]_DNA-U2 KC979049         379-TTTAATGAAGATATGGTTGGAATTCGCTGTCGTCGTCATGGAAGAAGAGTGA-441
nanoU2dir primer                                                    TGYAGAYRYCATGGAAGRA
```

Figure S1. Multialignment of nanovirus DNA-U2 sequences. Alignment of multiple sequences was performed with MultAlign (INRA, Toulouse, URL: http://multalin.toulouse.inra.fr/multalin/). Sequences displayed span nucleotides from the respective 5′- to 3′-coordinates indicated. The sequence of the degenerate primer nanoU2dir is shown below the alignment

```
FBNYV-[ES;R4]_DNA-U4 KC979034       601-ATTCAAAGGTTGATGAAGAAGATGTGGAGTATTTTAAACGGTTCTGGCAA-650
FBNYV-[ES;MU29D]_DNA-U4 KC979026    601-ATTCAAAGGTTGATGAAGAAGATGTGGAGTATTTTAAACGGTTCTGGCAA-650
FBNYV-[ES;R7]_DNA-U4 KC979042       601-ATTCAAAGGTTGATGAAGAAGATGTGGAGTATTTTAAACGGTTCTGGCAA-650
FBNYV-[AZ;13.5]_DNA-U4 KC979016     602-ATTCAAAGGTTGACGAAGAAGATGTGGAGTATTTCAAACGCTTCTGGCAA-651
FBNYV-[MA;23]_DNA-U4 GQ274024       602-ATTCAAAGGTTGATGAAGAAGATGTGGAGTATTTCAGACGGTTCTGGCAA-651
FBNYV-[SY;992-88]_DNA-U4 AJ749903   604-ATTCAAAGGTCGATGAAGAAGATGTGGAGTATTTAAAACGATTTTGGCAA-653
FBNYV-[EG;1-93]_DNA-U4 AJ749902     603-ACTCAAAGGTCGATGAAGAAGATGTGGAGTATTTCAGACGATTTTGGCAA-652
FBNYV-[AZ;12]_DNA-U4 KC979007       601-ATTCACACGTTGATGAAGATGATATGGAATATCTCAGACGGTTCTGGCAA-650
FBNSV-[AZ;1]_DNA-U4 KC978973        623-ACACAAAGGTTGAAGGTGACGATCTGGAATACTTGCAACGCCTATGGGAA-672
FBNSV-[AZ;10]_DNA-U4 KC978981       623-ACACGAAGGTTGAAGGTGATGATCTGGAATACTTGCAACGCCTATGGGAA-672
FBNSV-[MA;5]_DNA-U4 GQ274032        584-ACACGAAGGTTGAAGGAGATGATCTCGAATACTTGCAACGCCTATGGGAA-633
FBNSV-[ET;Hol-97]_DNA-U4 GU983873   614-AAACAAAGGTTGAAGGCGATGATCTTGAATATTTACAGCGATTGTGGGAA-663
FBNSV-[AZ;15]_DNA-U4 KC978999       620-ATACGAAGGTCGAAGGTGATGATCTAGACTATCTTAAACGTATGTGGGAA-669
FBYLV-[ET;231]_DNA-U4 HE654130      590-ATACGAAGGTGGACGATGAAGATTTGGAATATTTGCAACGTGTCTGGAAA-639
PYSV-[AT;15]_DNA-U4 KC979061        598-ACGTGAAGGTTGAAGGTGAAGATTTAGAGTATTTACAGTCTATATGGAGA-647
PNYDV-[DE;15]_DNA-U4 JN133285       598-ATGTCAAGGTTGAAGGTGAAGACCTGGAGTATTTACAGTCTTTATGGAAA-647
PNYDV-[AT;1]_DNA-U4 KC979050        597-ATGTCAAGGTTGAAGGTGAAGACCTGGAGTATTTACAGTCTTTATGGAAA-646
MDV-[JP;1]_DNA-U4 AB255373          576-ATACCTCTCTAGACGGTGAAGACCTTGAATATTTGCAACGTTTCTGGGAA-625
BMLRV-[AT;3]_DNA-U4 KC978947        585-ATATGAAGATTGAAGACGATGACCTTGAGTATCTTCAAGGAATATGGGAT-634
BMLRV-[SE;153]_DNA-U4 KC978965      576-ATATGAAGATTGAAGACGATGACCTTGAGTATCTTCAAGGAATATGGGAT-625
BMLRV-[AZ;47]_DNA-U4 KC978956       577-ATATGAAGATTGAAGACGATGACATGGAGTACATCAAAAGATTATGGGAG-626
nanoU4dir primer                               GGTTGAHGRHGAHGAYSTGGARTA
```

Figure S2. Multialignment of nanovirus DNA-U4 sequences. Alignment of multiple sequences was performed with MultAlign (INRA, Toulouse, URL: http://multalin.toulouse.inra.fr/multalin/). Sequences displayed span nucleotides from the respective 5′- to 3′-coordinates indicated. The sequence of the degenerate primer nanoU4dir is shown below the alignment.

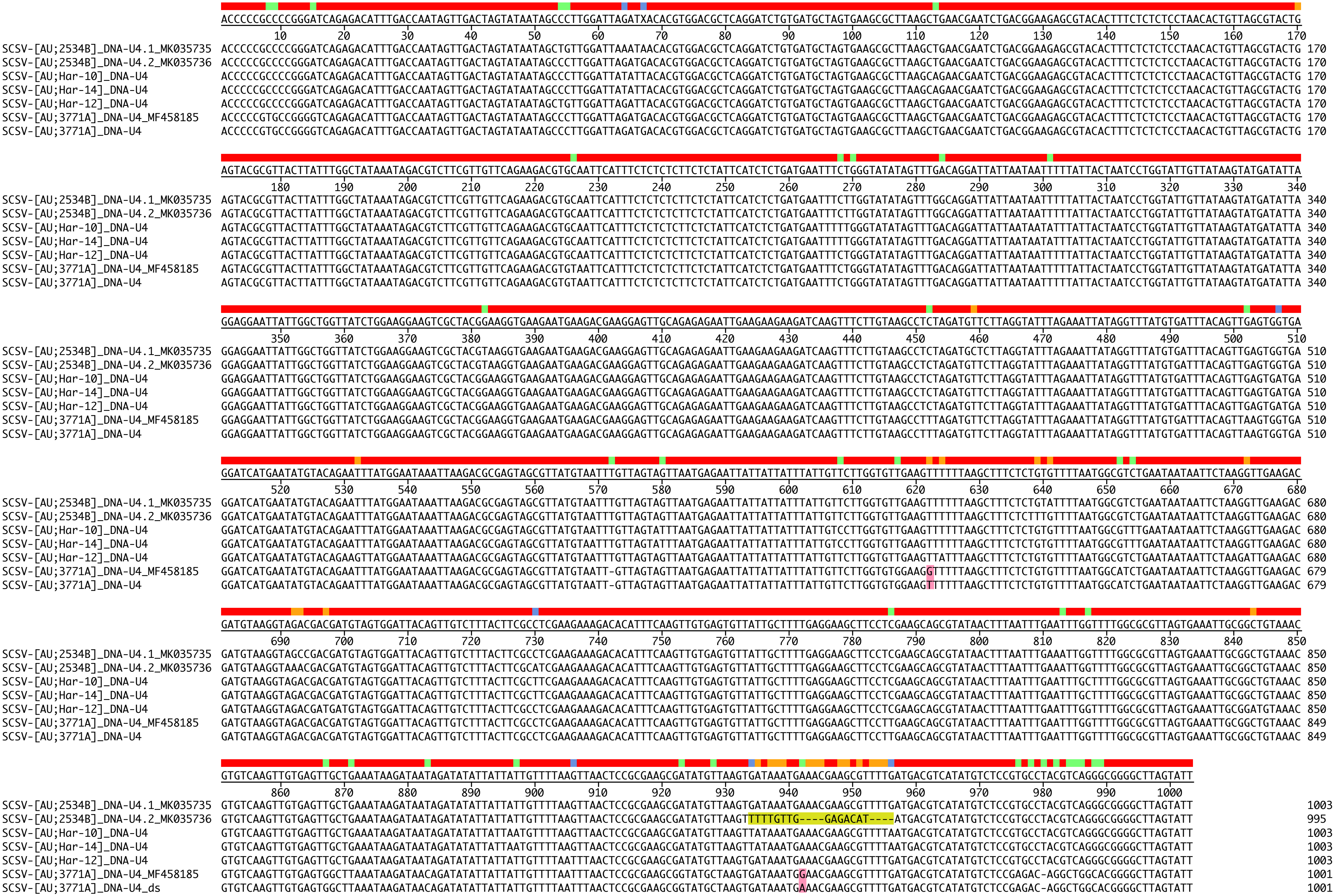

Figure S3. Multialignment of SCSV DNA-U4 sequences. Alignment was performed using MegAlign of DNA Star. The yellow box indicates sequence homologous to DNA-C. Pink boxes indicate nucléotide differences between two DNA-U4 sequences from SCSV-[AU;3771A], that of a cloned DNA (DNA-U4_MF458185) and that of the DNA-U4 consensus sequence determined by deep sequencing (DNA-U4_ds).

```
                                ACCCCCGTGCCGGGXTCAGAGACATTTGACCAATAGTTGACTAGTATAATAGCXXTTXGATTAXATGACACGTGGACGCTCAGGATCTGTGATGCTAGTGAAXCGCTTAAGCTGAACGAATCTGACGGAA
                                         10        20        30        40        50        60        70        80        90       100       110       120       130
SCSV-[AU;3771A]_DNA-M.1_MF458181 ACCCCCGTGCCGGGGTCAGAGACATTTGACCAATAGTTGACTAGTATAATAGCCCTTGGATTAGATGACACGTGGACGCTCAGGATCTGTGATGCTAGTGAAGCGCTTAAGCTGAACGAATCTGACGGAA 130
SCSV-[AU;3771A]_DNA-M.2_MK291271 ACCCCCGTGCCGGGATCAGAGACATTTGACCAATAGTTGACTAGTATAATAGCTGTTAGATTAAATGACACGTGGACGCTCAGGATCTGTGATGCTAGTGAATCGCTTAAGCTGAACGAATCTGACGGAA 130

                                GAGCGTTCACACTTAGATCTAGTTAGCGTACTTAGTACGCGTTGTCTTGGXTCTATAAATAGAGTGCTTCTGAACAGAXTGTTCAGAATTTCATAGCGAGATGGATTCTGGTGATGGTTACAATACATAC
                                        140       150       160       170       180       190       200       210       220       230       240       250       260
SCSV-[AU;3771A]_DNA-M.1_MF458181 GAGCGTTCACACTTAGATCTAGTTAGCGTACTTAGTACGCGTTGTCTTGG-TCTATAAATAGAGTGCTTCTGAACAGACTGTTCAGAATTTCATAGCGAGATGGATTCTGGTGATGGTTACAATACATAC 259
SCSV-[AU;3771A]_DNA-M.2_MK291271 GAGCGTTCACACTTAGATCTAGTTAGCGTACTTAGTACGCGTTGTCTTGGGTCTATAAATAGAGTGCTTCTGAACAGATTGTTCAGAATTTCATAGCGAGATGGATTCTGGTGATGGTTACAATACATAC 260

                                TCATATGAAGAAGGTGCTGGAGATGCGAAGAAGGAAGTTTTATATAAAATAGGTATTATTATGTTATGTATTGTAGGGATTGTAGTTTTATGGGTTTTAATTATATTATGTTGTGCTGTTCCTCGCTATG
                                        270       280       290       300       310       320       330       340       350       360       370       380       390
SCSV-[AU;3771A]_DNA-M.1_MF458181 TCATATGAAGAAGGTGCTGGAGATGCGAAGAAGGAAGTTTTATATAAAATAGGTATTATTATGTTATGTATTGTAGGGATTGTAGTTTTATGGGTTTTAATTATATTATGTTGTGCTGTTCCTCGCTATG 389
SCSV-[AU;3771A]_DNA-M.2_MK291271 TCATATGAAGAAGGTGCTGGAGATGCGAAGAAGGAAGTTTTATATAAAATAGGTATTATTATGTTATGTATTGTAGGGATTGTAGTTTTATGGGTTTTAATTATATTATGTTGTGCTGTTCCTCGCTATG 390

                                CTAAATCAACGATGGACGCTTGGTTATCTTCGTCTTCTATTATGAAGAGGAAGATGGCTTCAAGGATTACTGGTACTCCGTTTGAAGAAACTGGTCCTCATCGTGAAAGAAGATGGGCTGAAAGAAGAAC
                                        400       410       420       430       440       450       460       470       480       490       500       510       520
SCSV-[AU;3771A]_DNA-M.1_MF458181 CTAAATCAACGATGGACGCTTGGTTATCTTCGTCTTCTATTATGAAGAGGAAGATGGCTTCAAGGATTACTGGTACTCCGTTTGAAGAAACTGGTCCTCATCGTGAAAGAAGATGGGCTGAAAGAAGAAC 519
SCSV-[AU;3771A]_DNA-M.2_MK291271 CTAAATCAACGATGGACGCTTGGTTATCTTCGTCTTCTATTATGAAGAGGAAGATGGCTTCAAGGATTACTGGTACTCCGTTTGAAGAAACTGGTCCTCATCGTGAAAGAAGATGGGCTGAAAGAAGAAC 520

                                TGAAGCGACGAAXCAGAATAATAATGATAATGTAAATAGATTTAGTTGATATGTTGTAATTTTATATGGATTAATGAGAATTATTATTATTXTGTTCTTCGTCTGTGTTTTTTAAGCTTTTTCTGTGTTT
                                        530       540       550       560       570       580       590       600       610       620       630       640       650
SCSV-[AU;3771A]_DNA-M.1_MF458181 TGAAGCGACGAATCAGAATAATAATGATAATGTAAATAGATTTAGTTGATATGTTGTAATTTTATATGGATTAATGAGAATTATTATTATTATGTTCTTCGTCTGTGTTTTTTAAGCTTTTTCTGTGTTT 649
SCSV-[AU;3771A]_DNA-M.2_MK291271 TGAAGCGACGAACCAGAATAATAATGATAATGTAAATAGATTTAGTTGATATGTTGTAATTTTATATGGATTAATGAGAATTATTATTATTCTGTTCTTCGTCTGTGTTTTTTAAGCTTTTTCTGTGTTT 650

                                TAATGGCGTCTGGAGAGAGAAAGGAATAATTXTAAGGTTGAAGACGACGATAGTGCTTGACACTXTXCTTTXCTTCGTCTTGAAGCAAAGACAGCTGTCATTGCTTCGCCTCGAAGCAAGGTATCTACTT
                                        660       670       680       690       700       710       720       730       740       750       760       770       780
SCSV-[AU;3771A]_DNA-M.1_MF458181 TAATGGCGTCTGGAGAGAGAAAGGAATAATTGTAAGGTTGAAGACGACGATAGTGCTTGACACTTTACTTTGCTTCGTCTTGAAGCAAAGACAGCTGTCATTGCTTCGCCTCGAAGCAAGGTATCTACTT 779
SCSV-[AU;3771A]_DNA-M.2_MK291271 TAATGGCGTCTGGAGAGAGAAAGGAATAATTTTAAGGTTGAAGACGACGATAGTGCTTGACACTATTCTTTACTTCGTCTTGAAGCAAAGACAGCTGTCATTGCTTCGCCTCGAAGCAAGGTATCTACTT 780

                                TAATTGTAATTTGGTTTTGGCGCCTAAGGCGXGTCGCTAAAGAGTAAAXXAATAAAXTGACGTGGAGCGATATGATTGGTCATAATAAGCXAATAGATTTAATTGTTATTXTTTTXXTTTAACTCCGCGA
                                        790       800       810       820       830       840       850       860       870       880       890       900       910
SCSV-[AU;3771A]_DNA-M.1_MF458181 TAATTGTAATTTGGTTTTGGCGCCTAAGGCGCGTCGCTAAAGAGTAAAGAATAAACTGACGTGGAGCGATATGATTGGTCATAATAAGCCAATAGATTTAATTGTTATTGTTTTAATTTAACTCCGCGA 909
SCSV-[AU;3771A]_DNA-M.2_MK291271 TAATTGTAATTTGGTTTTGGCGCCTAAGGCGTGTCGCTAAAGAGTAAAGCAATAAAATGACGTGGAGCGATATGATTGGTCATAATAAGCTAATAGATTTAATTGTTATTCTTTTG-TTTAACTCCGCGA 909

                                AGCGGTATGXTXAGTGAXXAXXGXAXXGXAXXXGXGTXTTXXXXXXXXXGATGACGTCATATGTCTCCGXGACAGGCTGGCACGGGGCTTAGTATT
                                        920       930       940       950       960       970       980       990      1000
SCSV-[AU;3771A]_DNA-M.1_MF458181 AGCGGTATGCTAAGTGATAAATGAAACGAA---GCGTTTT---------GATGACGTCATATGTCTCCGAGACAGGCTGGCACGGGGCTTAGTATT                                  993
SCSV-[AU;3771A]_DNA-M.2_MK291271 AGCGGTATGTTTAGTGAGGATAGGATTGTAATTGTGTATTCGTGAGTGAGATGACGTCATATGTCTCCGCGACAGGCTGGCACGGGGCTTAGTATT                                  1005
```

Figure S4. SCSV-[AU;3771A]_DNA-M.1_MF458181_vs_DNA-M.2_MK291271 alignment. Alignment was performed using MegAlign of DNA Star. The yellow box indicates sequence homologous to DNA-R.

```
                              ACXCCCGTGCCGGGATCAGAGACATTTGACTAAATGTTGACTTGGAATAATAGCCCTTGGATTAGATGACACGTGGACGCTCAGGATCTGTGATGCTAGTGAAGCGCTTAAGCTGAACGAATCTGACGGA
                                       10        20        30        40        50        60        70        80        90       100       110       120       130
SCSV-[AU;F]_DNA-N_U16733      AC-CCCGTGCCGGGATCAGAGACATTTGACTAAATGTTGACTTGGAATAATAGCCCTTGGATTAGATGACACGTGGACGCTCAGGATCTGTGATGCTAGTGAAGCGCTTAAGCTGAACGAATCTGACGGA 129
SCSV-[AU;F*]_DNA-N_MK291272   ACCCCCGTGCCGGGATCAGAGACATTTGACTAAATGTTGACTTGGAATAATAGCCCTTGGATTAGATGACACGTGGACGCTCAGGATCTGTGATGCTAGTGAAGCGCTTAAGCTGAACGAATCTGACGGA 130

                              AGAGCGGACAAACGCACATGGACTATGGCCCACTGCTTTATTAAAGAAGTGAATGACAGCTGTCTTTGCTTCAAGACGAAGTAAAGAATAGTGGAAAACGCGTAAAGAATAAGCGTACTCAGTACGCTTC
                                      140       150       160       170       180       190       200       210       220       230       240       250       260
SCSV-[AU;F]_DNA-N_U16733      AGAGCGGACAAACGCACATGGACTATGGCCCACTGCTTTATTAAAGAAGTGAATGACAGCTGTCTTTGCTTCAAGACGAAGTAAAGAATAGTGGAAAACGCGTAAAGAATAAGCGTACTCAGTACGCTTC 259
SCSV-[AU;F*]_DNA-N_MK291272   AGAGCGGACAAACGCACATGGACTATGGCCCACTGCTTTATTAAAGAAGTGAATGACAGCTGTCTTTGCTTCAAGACGAAGTAAAGAATAGTGGAAAACGCGTAAAGAATAAGCGTACTCAGTACGCTTC 260

                              GTGGCTTTATAAATAGTGCTTCGTCTXXXTXTTXXXXXXXXXXXXXXXXXXXXXXTTAAGCTTTGTTCTGCGTTTTAATGGCGGACTGGTTTCACAGTGCGCTTAAGACATGTACTCATGTCTGTGATTT
                                      270       280       290       300       310       320       330       340       350       360       370       380       390
SCSV-[AU;F]_DNA-N_U16733      GTGGCTTTATAAATAGTGCTTCGTCTTATTCTTCGTTGTATCATCAACGAAGAAGTTAAGCTTTGTTCTGCGTTTTAATGGCGGACTGGTTTCACAGTGCGCTTAAGACATGTACTCATGTCTGTGATTT 389
SCSV-[AU;F*]_DNA-N_MK291272   GTGGCTTTATAAATAGTGCTTCGTCTGTGTTTT----------------------TTAAGCTTTGTTCTGCGTTTTAATGGCGGACTGGTTTCACAGTGCGCTTAAGACATGTACTCATGTCTGTGATTT 368

                              TTCAGATATTAAGGCGTCTTCACAACAGGATTTCTTCTGTTGTGATAGTATGCGAGGTAAATTATCTGAACCTAGGAAGGTGTTGTTAGTTAGTTGTTTTGTAAGTTTTACTGGTAGTTTTTATGGAAGT
                                      400       410       420       430       440       450       460       470       480       490       500       510       520
SCSV-[AU;F]_DNA-N_U16733      TTCAGATATTAAGGCGTCTTCACAACAGGATTTCTTCTGTTGTGATAGTATGCGAGGTAAATTATCTGAACCTAGGAAGGTGTTGTTAGTTAGTTGTTTTGTAAGTTTTACTGGTAGTTTTTATGGAAGT 519
SCSV-[AU;F*]_DNA-N_MK291272   TTCAGATATTAAGGCGTCTTCACAACAGGATTTCTTCTGTTGTGATAGTATGCGAGGTAAATTATCTGAACCTAGGAAGGTGTTGTTAGTTAGTTGTTTTGTAAGTTTTACTGGTAGTTTTTATGGAAGT 498

                              AATAGGAATGTTAGAGGTCAAGTTCAGTTGGGTATGCAGCAAGATGATGGCGTTGTTCGTCCAATAGGATATATTCCTATTGGGGGTTATTTGTATCATGATGATTATGGATATTATCAAGGAGAGAAGA
                                      530       540       550       560       570       580       590       600       610       620       630       640       650
SCSV-[AU;F]_DNA-N_U16733      AATAGGAATGTTAGAGGTCAAGTTCAGTTGGGTATGCAGCAAGATGATGGCGTTGTTCGTCCAATAGGATATATTCCTATTGGGGGTTATTTGTATCATGATGATTATGGATATTATCAAGGAGAGAAGA 649
SCSV-[AU;F*]_DNA-N_MK291272   AATAGGAATGTTAGAGGTCAAGTTCAGTTGGGTATGCAGCAAGATGATGGCGTTGTTCGTCCAATAGGATATATTCCTATTGGGGGTTATTTGTATCATGATGATTATGGATATTATCAAGGAGAGAAGA 628

                              CGTTCAATCTGGACATCGAGTCAGATTATCTGAAGCCTGATGAAGATTTTTGGAAGAGATTTACAATTAATATTGTAAATGATXAAGGATTAGATGATAGGTGTGATGTAAAATGTTATGTAGTTCATAC
                                      660       670       680       690       700       710       720       730       740       750       760       770       780
SCSV-[AU;F]_DNA-N_U16733      CGTTCAATCTGGACATCGAGTCAGATTATCTGAAGCCTGATGAAGATTTTTGGAAGAGATTTACAATTAATATTGTAAATGATAAAGGATTAGATGATAGGTGTGATGTAAAATGTTATGTAGTTCATAC 779
SCSV-[AU;F*]_DNA-N_MK291272   CGTTCAATCTGGACATCGAGTCAGATTATCTGAAGCCTGATGAAGATTTTTGGAAGAGATTTACAATTAATATTGTAAATGATCAAGGATTAGATGATAGGTGTGATGTAAAATGTTATGTAGTTCATAC 758

                              GATGCGTATTAAGGTGTAATTGTTATTATCAATAAAAGAATTTTTATTGTTATTGTGTTATTTGGTAATTTATGCTTATAAGTAATTCTATGATTAATTGTGAATTAATAAGACTXATXAXXGXATAAXX
                                      790       800       810       820       830       840       850       860       870       880       890       900       910
SCSV-[AU;F]_DNA-N_U16733      GATGCGTATTAAGGTGTAATTGTTATTATCAATAAAAGAATTTTTATTGTTATTGTGTTATTTGGTAATTTATGCTTATAAGTAATTCTATGATTAATTGTGAATTAATAAGACTAATGA--GGATAATA 907
SCSV-[AU;F*]_DNA-N_MK291272   GATGCGTATTAAGGTGTAATTGTTATTATCAATAAAAGAATTTTTATTGTTATTGTGTTATTTGGTAATTTATGCTTATAAGTAATTCTATGATTAATTGTGAATTAATAAGACTGATTAATGAATAAAG 888

                              AXTXXXTTTXATTXXXXXAXTXACTCXGCGAAGCXXTXTGTXXTXXTXXXGTXXGAGXCAXXTGAXXXXXXXTGTCTCCGCGACAGGCTGGCACGGGGCTTAGTATT
                                      920       930       940       950       960       970       980       990      1000      1010
SCSV-[AU;F]_DNA-N_U16733      ATTGAATTTGATTA---AATTAACTCTGCGAAGCTATATGTCTT--TCACGTGAGAGTCACGTGA-------TGTCTCCGCGACAGGCTGGCACGGGGCTTAGTATT                      1002
SCSV-[AU;F*]_DNA-N_MK291272   AGTTGTTTTTATTCTTTGAATTACTCCGCGAAGCGGTGTGTTATGTTTTTGTTGGAGACATATGACGTCATATGTCTCCGCGACAGGCTGGCACGGGGCTTAGTATT                      995
```

Figure S5. SCSV-[AU;F]_DNA-N_U16733 vs SCSV-[AU;F*]_DNA-N_MK291272 alignment. Alignment was performed using MegAlign of DNA Star. The yellow box indicates sequence homologous to DNA-S.

Table S1. Primers used for amplification of SCSV DNAs

DNA	Primer name (direction)	Sequence (5'-3')	Tm, C°	Use [a, b]
nanovirus DNAs [c]	nano-STL-dir	GGCTTAGATATTACCCC	49.2	2
	nano-STL-rev	GGTAATACTAAGCCCC	49.2	2 [f]
nanovirus DNA-U2 [d]	nano-U2-dir	TGYAGAYRYCATGGAAGRA	53.4	2 [f]
nanovirus DNA-U4 [e]	nano-U4-dir	GGTTGAHGRHGAHGAYSTGGARTA	61.8	2 [f]
R	SCSV-R-dir	TTCGTCTACAGTCCTTCGTCTCCATCAA	65.1	1, 4
	SCSV-R-rev	GTAGACGAATAATTTGGGTGTATGGCCC	65.1	1, 4
	SCSV-R790-dir	GAATACGTCAATTATGGAGTCATTGAACAGCT	64.4	1
	SCSV-R790-rev	ACGTATTCCTCGAAGCTTCGAGGAAAGTCG	65.5	1
M	SCSV-M-dir	GAAGATGGGCTGAAAGAAGAACTGAAGCG	66.7	1, 4
	SCSV-M-rev	CCATCTTCTTTCACGATGAGGACCAGTTTC	66.8	1, 4
	SCSV-M390-dir	CTAAATCAACGATGGACGCTTGGTTATC	63.7	1
	SCSV-M390-rev	TGATTTAGCATAGCGAGGAACAGCACAAC	65.3	1
N	SCSV-N-dir	GGAAGGTGTTGTTAGTTAGTTGTTTTGTAA	61.3	1, 4
	SCSV-N-rev	CACCTTCCTAGGTTCAGATAATTTACCTCG	65.4	1, 4
	SCSV-N420-dir	GCTTCGTGGCTCTATAAATAGTGCTTCGTCTT	63.4	1
	SCSV-N420-rev	CACGAAGCGTACTGAGTACGCTTATTCTTTACG	65.0	1
C	SCSV-C-dir	GAGTTATGGAGGAGATGCATTACAAGCTT	63.9	1, 4
	SCSV-C-rev	CCATAACTCTCCATTTCAGATTTA	55.9	1, 4
S	SCSV-S-dir	GGAGGTTAAGCCATTCGCTGATGGTTCAAG	68.1	1, 4
	SCSV-S-rev	TAACCTCCGCTCCAGTAACATCTCTCTTAT	65.4	1, 4
U1	SCSV-U1-dir	GTGATTAACGTGAAGGTACTGAGGTT	61.6	1, 4
	SCSV-U1-rev	CGTTAATCACTTGAACCTTCTC	56.5	1, 4
U2	SCSV_37-U2frDir	CTAAGATGCCTAGAGATGGACCTGCAAGGTG	69.5	1
	SCSV_37-U2frRev	CTTAGGATAAGCCTTCACTCTTCTTCCATG	65.4	1
	SCSV_37-U2fr2dir	AATTATAGATATCAAGTGTATTACTCTG	56.3	1, 4
	SCSV_37-U2fr2rev	CTATAATTACCATAATCATACAAATCA	54.3	1, 4
	SCSV-U2-224-dir	GATTTGTTCTGGGTTTTATATGAA	55.3	1
	SCSV-U2-224-rev	CAGAACAAATCTTGTTCTTCCTT	54.2	1
U4	SCSV_37-U4fr2dir	GTGAGGATCATGAATATGTACAG	57.1	1, 4
	SCSV_37-U4fr2rev	TCCTCACCACTTAACTGTAAATCAC	59.7	1, 4
	SCSV-U4-340-dir	GGAGGAATTATTGGCTGGTTATCTGGAA	63.7	1,3, 4
	SCSV-U4-340-rev	ATTCCTCCTAATATCATACTTATAACAATACCAGG	63.6	1, 4
	SCSV-U4.1-932-rev	CGTTTCATTTATCACTTAACATATCGC	58.9	3
	SCSV-U4.2-932-rev	TCTCCAACAAAAACTTAACATATCGC	58.5	3

a – Primers used for PCR amplification of the corresponding DNAs with the purpose of: (1) entire component amplification and cloning, (2) partial amplification, (3) specific amplification of DNA-U4.1 or DNA-U4.2 fragment by PCR, (4) detection of SCSV DNAs in field samples of symptomatic subterranean clover.

b – PCR amplifications employing component-specific primers were performed at annealing temperatures corresponding to a minimal Tm of a given primer pair.

c – Primers designed based on the DNA sequence in the region with potential stem-loop structure (STL) of nanovirus DNAs

d – Primers designed on the basis of the alignment of the nanovirusDNA-U2 sequences.

e - Primers designed on the basis of the alignment of the nanovirus DNA-U4 sequences.

f - PCR amplifications using nano-STL-rev in combination with a degenerate primer were performed with Taq II DNA polymerase as follows: 95° C, 5 min; 5 cycles of: 95° C, 30 s, 45° C, 45 s, 72° C, 2 min; followed by 35 cycles of: 95° C, 30 s, gradient 45° C to 65° C, 45 s, 72° C, 45 s; final elongation: 72° C, 4 min.

Table S2. Summary of deep sequencing data from three SCSV samples

	Sample / DNA	SCSV-[AU;3771A] (1,519,544 total reads)			SCSV-[AU; 2534B] (1,560,076 total reads)			SCSV-[AU;-F*] (1,984,716 total reads)		
		Reads	% of reads	Cov. Mean	Reads	% of reads	Cov. Mean	Reads	% of reads	Cov. Mean
Genomic DNAs	DNA-R	4,273	0.28	6,878	32,450	2.08	6,156	26,548	1.34	4,881
	DNA-S	10,419	0.69	1,849	73,136	4.69	14,378	33,825	1.70	6,496
	DNA-M	27,366	1.80	4,394	68,165	4.37	12,132	400,292	20.17	72,184
	DNA-M.2 [a]	12,979	0.85	2,074						
	DNA-N	11,943	0.79	2,072	100,037	6.41	19,303	12,764	0.64	2,343
	DNA-C	20,471	1.35	3,602	43,620	2.80	8,152	6,168	0.31	796
	DNA-U1	5,727	0.38	929	40,129	2.57	7,436	121,430	6.12	22,534
	DNA-U2	2,510	0.17	421	41,474	2.66	7,863	2,513	0.13	431
	DNA-U4	86,676	5.70	14,167	109,090	6.99	19,932			
	DNA-U4.2				123,375	7.91	22,640			
alphasatellites	SCSA 1	169,370	11.15	29,851	293,801	18.83	57,449			
	SCSA 1.1							91,250	4.60	17,207
	SCSA 1.2							93,260	4.70	17,178
	SCSA 2	125,028	8.23	22,230	192,094	12.31	38,112	460,008	23.18	89,133
	SYSA 3	31,974	2.10	5,520						
	total	508,736	33.48		1,117,371	71.62		1,248,058	62.88	

Circularized consensus sequences were generated from *de novo* assembly of deep sequencing reads.
Cov. Mean – average coverage.
a – recombinant with DNA-R

Table S3. Nucleotide substitutions

Comparison of two different SCSV isolates	Total changes	% divergence	C/A	G/A	T/A	G/C	T/C	T/G	indel	other
SCSV-[AU;3771A]_DNA-R_MF458178<br>SCSV-[AU;2534B]_DNA-R_MK035728	12	1,2	1	3	1	1	4	2	0	
SCSV-[AU;3771A]_DNA-S_MF458179<br>SCSV-[AU;2534B]_DNA-S_MK035729	38	3,8	1	8	8	4	11	5	1	
SCSV-[AU;3771A]_DNA-C_MF458180<br>SCSV-[AU;2534B]_DNA-C_MK035730	21	1,7	0	5	4	2	3	3	4	
SCSV-[AU;3771A]_DNA-M_MF458181<br>SCSV-[AU;2534B]_DNA-M_MK035731	50	3,7	2	6	8	6	10	4	14	
SCSV-[AU;3771A_DNA-N_MF458182<br>SCSV-[AU;2534B]_DNA-N_MK035732	11	1	1	4	1	0	2	2	1	
SCSV-[AU;3771A]_DNA-U1_MF458183<br>SCSV-[AU;2534B]_DNA-U1_MK035733	26	2,5	2	9	2	4	5	2	2	
SCSV-AU;3771A]_DNA-U2_MF458184<br>SCSV-[AU;2534B]_DNA-U2_MK035734	15	1,4	1	4	1	2	3	3	1	
SCSV-[AU;3771A]_DNA-U4_MF458185<br>SCSV-[AU;2534B]_DNA-U4_MK035735	38	3,7	2	11	2	4	10	7	2	
**Sum**	**211**		**10**	**50**	**27**	**23**	**48**	**28**	**25**	

Comparison of cloned DNAs vs deep sequenced DNAs (_ds) of the same isolate (SCSV-[3771A])	Total changes	% divergence	C/A	G/A	T/A	G/C	T/C	T/G	indel	other
SCSV-[AU;3771A]_DNA-R_MF458178<br>SCSV-[AU;3771A]_DNA-R_ds	0	0	0	0	0	0	0	0	0	
SCSV-[AU;3771A]_DNA-S_MF458179<br>SCSV-[AU;3771A]_DNA-S_ds	5	0,5	0	0	1	0	3	1	0	
SCSV-[AU;3771A]_DNA-C_MF458180<br>SCSV-[AU;3771A]_DNA-C_ds	7	0,7	0	1	0	1	3	2	0	
SCSV-[AU;3771A]_DNA-M_MF458181<br>SCSV-[AU;3771A]_DNA-M_ds	2	0,2	0	0	0	0	0	2	0	
SCSV-[AU;3771A]_DNA-M_MF458181<br>SCSV-[AU;3771A]_DNA-M.2_ds (recombinant with DNA-R)	25	3,7	2	6	3	4	5	2	2 + 12†	† part of 32 nt derived from DNA-R
SCSV-[AU;3771A_DNA-N_MF458182<br>SCSV-[AU;3771A_DNA-N_ds	8	0,8	1	3	1	0	2	1	0	
SCSV-[AU;3771A]_DNA-U1_MF458183<br>SCSV-[AU;3771A]_DNA-U1_ds	15	1,4	1	5	1	3	3	1	1	
SCSV-AU;3771A]_DNA-U2_MF458184<br>SCSV-AU;3771A]_DNA-U2_ds	0	0	0	0	0	0	0	0	0	
SCSV-[AU;3771A]_DNA-U4_MF458185<br>SCSV-[AU;3771A]_DNA-U4_ds	2	0,2	0	1	0	0	0	1	0	
**Sum, counting recombination event as 1**	**64**		**4**	**16**	**6**	**8**	**16**	**10**	**1**	

Comparison of published SCSV-[AU;F] DNAs with deep sequenced SCSV-[AU;F*] progeny	Total changes	% divergence	C/A	G/A	T/A	G/C	T/C	T/G	indel	other
SCSV-[AU;F]_DNA-R_AJ290434<br>SCSV-[AU;F*]_DNA-R	0	0	0	0	0	0	0	0	0	
SCSV-[AU;F]_DNA-S_U16734<br>SCSV-[AU;F*]_DNA-S	12	1	1	1	2	1	2	3	2	
SCSV-[AU;F]_DNA-C_U16732<br>SCSV-[AU;F*]_DNA-C	3	0,3	0	0	0	2	1	0	0	
SCSV-[AU;F]_DNA-M_U16730<br>SCSV-[AU;F*]_DNA-M	29	1,8	2	4	1	2	6	3	11	
SCSV-[AU;F]_DNA-N_U16733<br>SCSV-[AU;F*]_DNA-N (Δ 22nt & 87nt recombinant with DNA-S)	36	3,6	2	9	8	0	6	8	1 + Δ22	87 nt recombinant with DNA-S
SCSV-[AU;F]_DNA-U1_U16736<br>SCSV-[AU;F*]_DNA-U1	11	0,8	1	3	1	0	3	0	3	
SCSV-[AU;F*]_DNA-U2_MF458186 (cloned DNA)<br>SCSV-[AU;F*]_DNA-U2	3	0,3	2	1	0	0	0	0	0	
SCSA1-[AU;F]_alphasat_1_U16731<br>SCSA1-[AU;F*]_alphasat_1.1	3	0,3	0	0	1	0	1	1	0	
SCSA1-[AU;F]_alphasat_1_U16731<br>SCSA1-[AU;F*]_alphasat_1.2 (Δ24 nt)	4	0,3	1	0	1	0	1	0	Δ 24	
SCSA2-[AU;F]_alphasat_2_U16735<br>SCSA2-[AU;F*]_alphasat_2	1	0,1	1	0	0	0	0	0	0	
**Sum, counting deletions >20 nt and recobination event as 1**	**102**		**10**	**18**	**14**	**5**	**20**	**15**	**16**	

Table S5. Detection by PCR of SCSV DNAs in field samples of symptomatic subterranean clover

DNA / Sample	-R	-S		-M		-C		-N	-U1		-U2		-U4[a]		-U4[b]	
	x35[c]	x35	x43[d]	x35	x43	x35	x43	x43	x35	x43	x35	x43	x35	x43	x35	x43
1	-	-	nt[e]	-	nt	-	nt	-	-	nt	-	-	-	-	-	-
2	-	-	nt	-	nt	-	nt	-	-	nt	-	-	-	-	-	-
3	++	+	++	++	nt	-	++	+	+	nt	+	+	-/+	++	-	-/+
4	++	-	+	-	+	-	++	++	-	++	-	+	-	-	-	-
5	++	-	++	-	+	-	-/+	-	-	-/+	-	+/-	-	-	-	-
6	+++	+++	nt	+++	nt	+++	nt	+++	+++	nt	+++	+++	+++	+++	+	++
7	++	+	nt	++	nt	++	nt	+	+/-	nt	+	+	-/+	+	-/+	-/+
8	-	-	nt	-	nt	-	nt	-	-	nt	-	-	-	-	-	-
9	-	-	nt	-	nt	-	nt	-	-	nt	-	-	-	-	-	-
10	++	-	++	++	nt	-	++	+	-	++	+	++	-	+	+	-/+
11	++	+	nt	++	nt	-	-	+	+	nt	+	++	-/+	++	-/+	+
12	++	++	nt	++	nt	++	nt	++	++	nt	+	++	+	+	+	+
13	++	-	-	-	++	-	-	-	-	+/-	-	+	-	+/-	-	-
14	++	++	nt	++	nt	++	nt	++	++	nt	++	++		++	+	+
Total SCSV-positive samples	10	9		10		8		8	10		10		8		7	

Subterranean clover plants showing symptoms of potential SCSV infection were collected on December 24, 2018, in Harrison, ACT.
After total DNA extractions, PCR reactions were performed using primers specific for SCSV DNA (indicated in Table S1), the amplified DNAs were subjected to agarose gel electrophoresis. The relative amounts of amplification products from different samples were estimated visually by staining intensity, ranging from traces (-/+) to very high amplification levels (+++).
a – primers SCSV_37-U4fr2dir and SCSV_37-U4fr2rev.
b – primers SCSV-U4-340-dir and SCSV-U4-340-rev.
c - 35 cycles of PCR amplification.
d - 43 cycles of PCR amplification.
e - if 35 PCR cycles yielded amplified DNA of a given genome component, no amplification using 43 cycles was done.

Table S6. Detection of SCSV DNAs in field samples after rolling circle amplification followed by PCR.

DNA / Sample	-S	-C	-N	-U4[b]
1	+++	+++	+++	+++
4	+++	+++	+++	+++
5	+++	+++	+++	+++
11	+++	+++	+++	+++
13	+++	+++	+++	+++

Total DNA extractions of samples 1, 4, 5, 11 and 13 (same as in Table S5) were subjected to rolling circle amplification followed by PCR amplification (35 cycles) using the same SCSV component-specific primers as in Table S5.
